# Supplementary material for: Convergent evolution in Afrotheria and non-afrotherians demonstrates high evolvability of the mammalian inner ear
Source: Nat Commun. 2024 Sep 16;15:7869. doi: 10.1038/s41467-024-52180-1 (PMC11405882; doi:10.1038/s41467-024-52180-1)
Supplement: Supplementary file 1 — Supplementary Information [file 41467_2024_52180_MOESM1_ESM.pdf]

Supplementary Information for

"Convergent evolution in Afrotheria and non-afrotherians demonstrates high evolvability of the mammalian inner ear"

Nicole D.S. Grunstra, Fabian Hollinetz, Guillermo Bravo Morante, Frank E. Zachos, Cathrin Pfaff, Viola Winkler, Philipp Mitteroecker & Anne Le Maître

CONTENTS

Supplementary Figures

- Suppl. Figs 1 and 2: Principal Component Analysis (PCA)
- Suppl. Figs 3 and 4: Phylogenetic Partial Least Squares (phylo PLS)
- Suppl. Figs 5 and 6: Jackknife of the Procrustes distances
- Suppl. Fig 7: *K*-fold cross-validation of the PLS analyses

Supplementary Tables

- Suppl. Table 1: example of computation of pairwise Procrustes distances

Supplementary Notes

- Note 1: Further details on pairwise Procrustes distance computation
- Note 2: Principal Component Analysis (PCA)
- Note 3: Phylogenetic Partial Least Squares (phylo PLS)
- Note 4: Jackknife of the Procrustes distances
- Note 5: *K*-fold cross-validation of the PLS analyses

Supplementary Figures

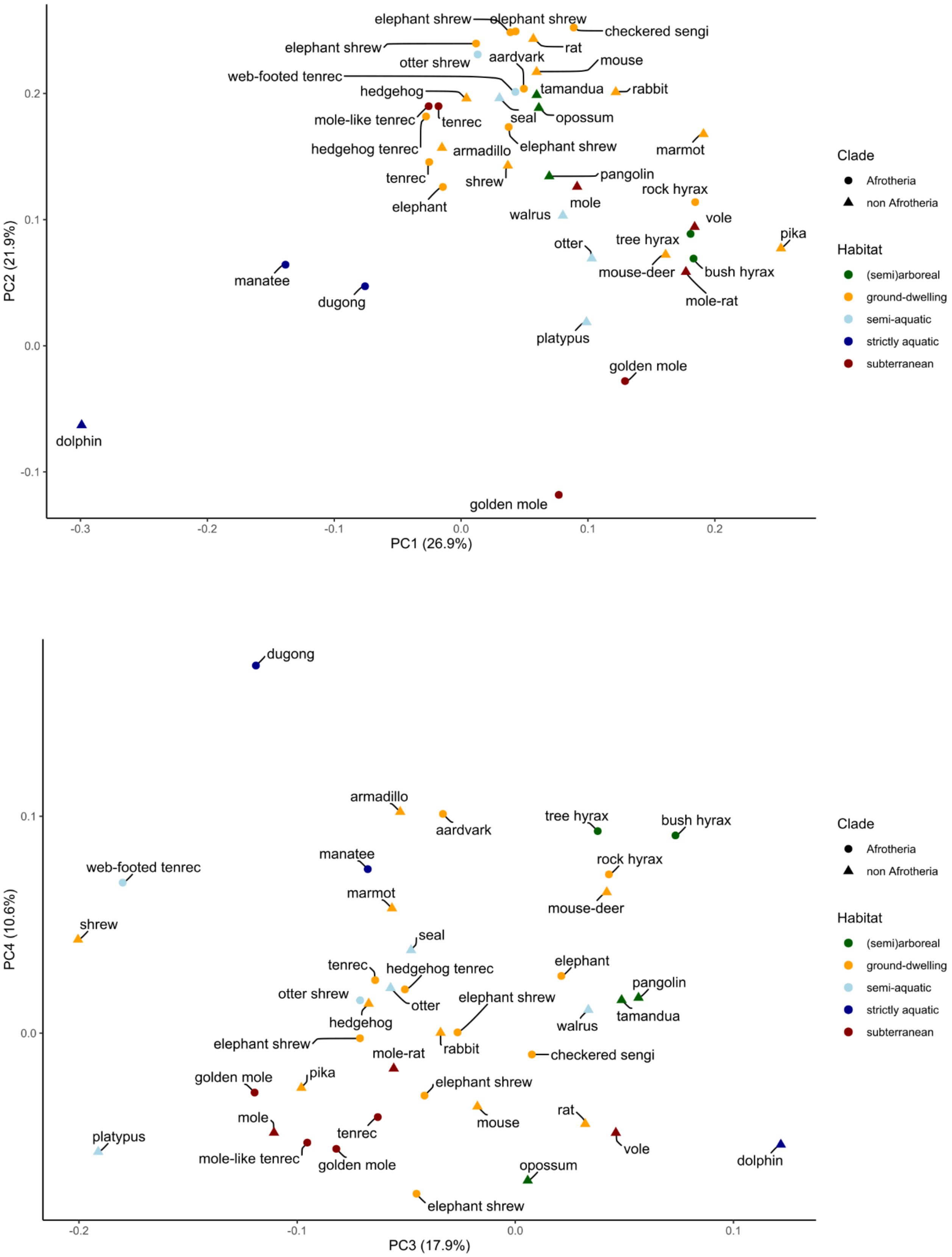

Supplementary Figure 1: Scores for the first four principal components of the Procrustes shape coordinates of the bony labyrinth. Shown are PCs 1 and 2 (top) and 3 and 4 (bottom). The scores are colour-coded to correspond to five broadly defined habitat groups for visualisation of a functional signal; group assignment was not used in the computation of the scores. Note that when a species' common name appears several times, it corresponds to closely related and morphologically and/or ecologically similar species (e.g., golden moles). Source data are provided in the Source Data file.

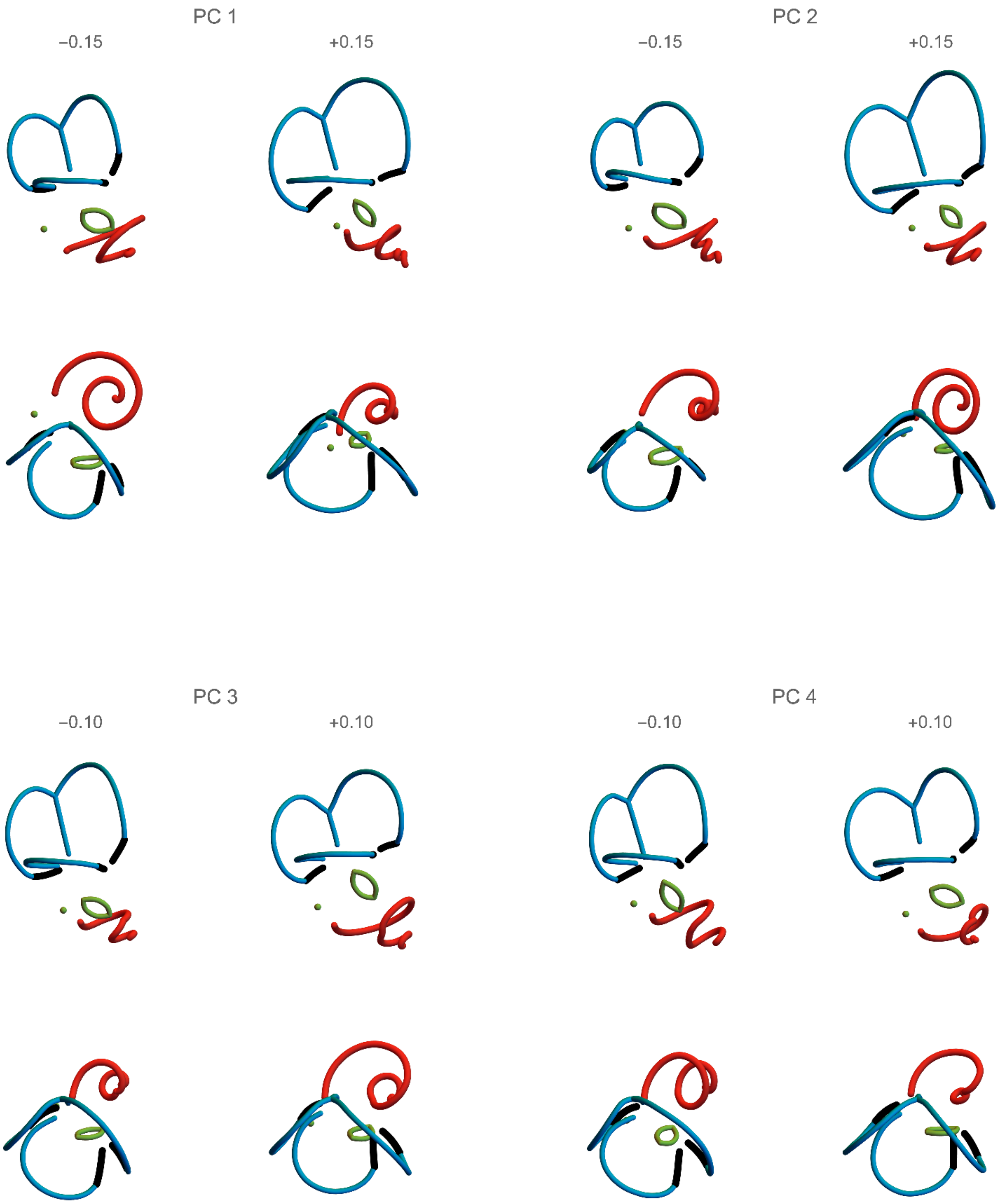

Supplementary Figure 2: Shape patterns corresponding to the first four principal components (PC). Shape patterns are shown in lateral and superior views (top and bottom rows for each PC, respectively). The semicircular canals are in blue, the cochlea is in red, and the vestibular (oval) and cochlear (point) windows are in green; the different ear parts correspond to the view in Fig. 2b and are also labelled in Figs 3 and 4 in the main article . Source data are provided in the Source Data file.

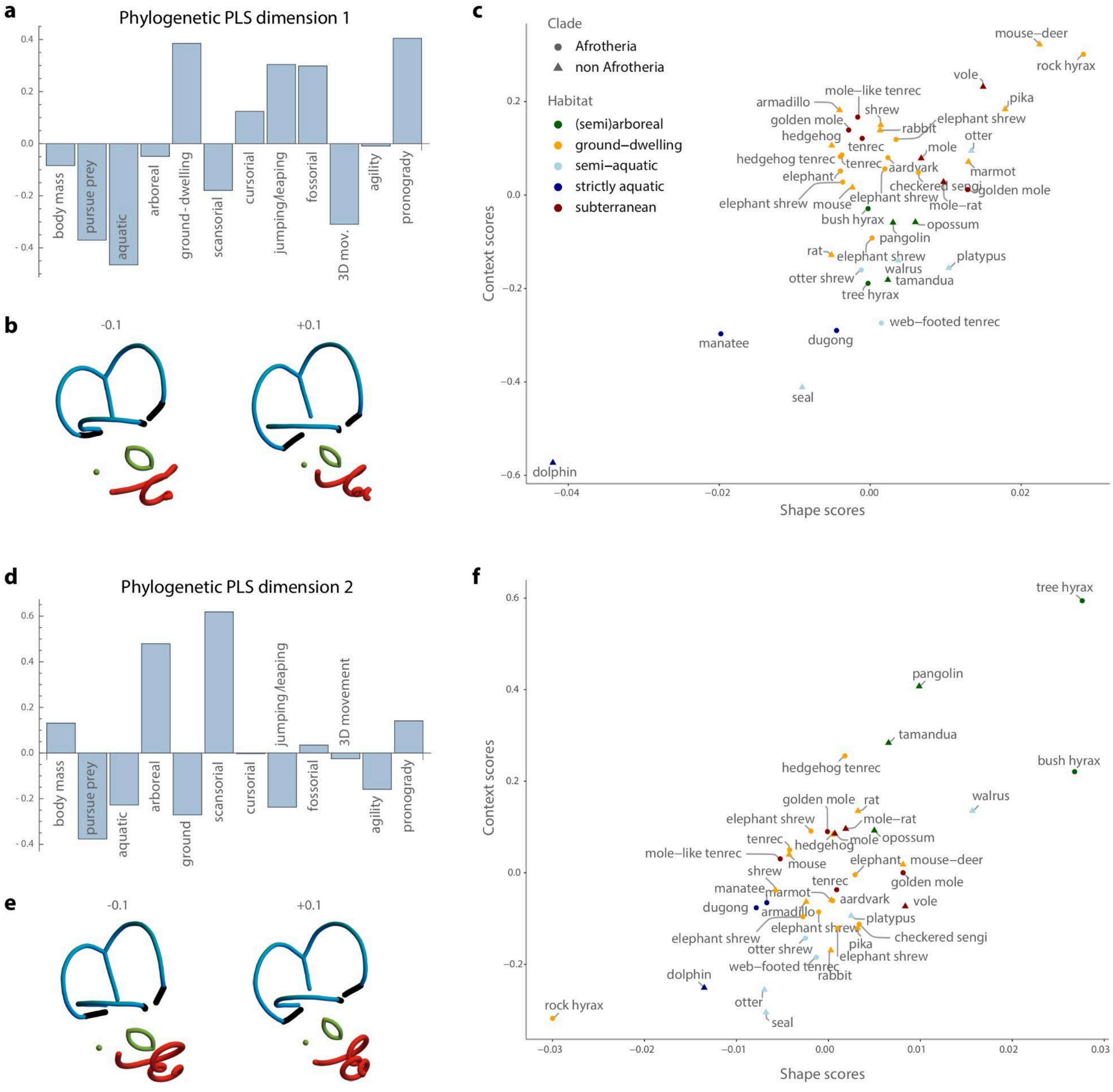

Supplementary Figure 3: Results for the phylogenetic partial least squares analysis of bony labyrinth shape and the contextual variables (PLS 1 and 2). First two dimensions of the phylogenetic two-block partial least squares (2B-PLS) analysis between the Procrustes shape coordinates of the bony labyrinth and the 12 contextual variables. **a** and **d** Loadings for the first and second phylogenetic PLS dimensions that represent the contextual patterns with highest and next highest covariance, respectively. **b** and **e** Loadings for the first and second phylogenetic PLS dimensions that represent the shape patterns with highest and next highest covariance, showing the semicircular canals in blue, the cochlea in red, and the vestibular (oval) and cochlear (point) windows in green; the different ear parts are also labelled in Figs. 3 and 4 in the main text.. The orientation of the bony labyrinths is the same as in Fig. 2b. **c** and **f** Scatterplots of the corresponding contextual and shape scores for phylogenetic PLS 1 and PLS 2. The colour code corresponds to five habitat types which were assigned to species as a visual aid only; this grouping was not used for computing the phylogenetic PLS. Note that when a species' common name appears several times, it corresponds to different species. Source data is provided in the Source Data file.

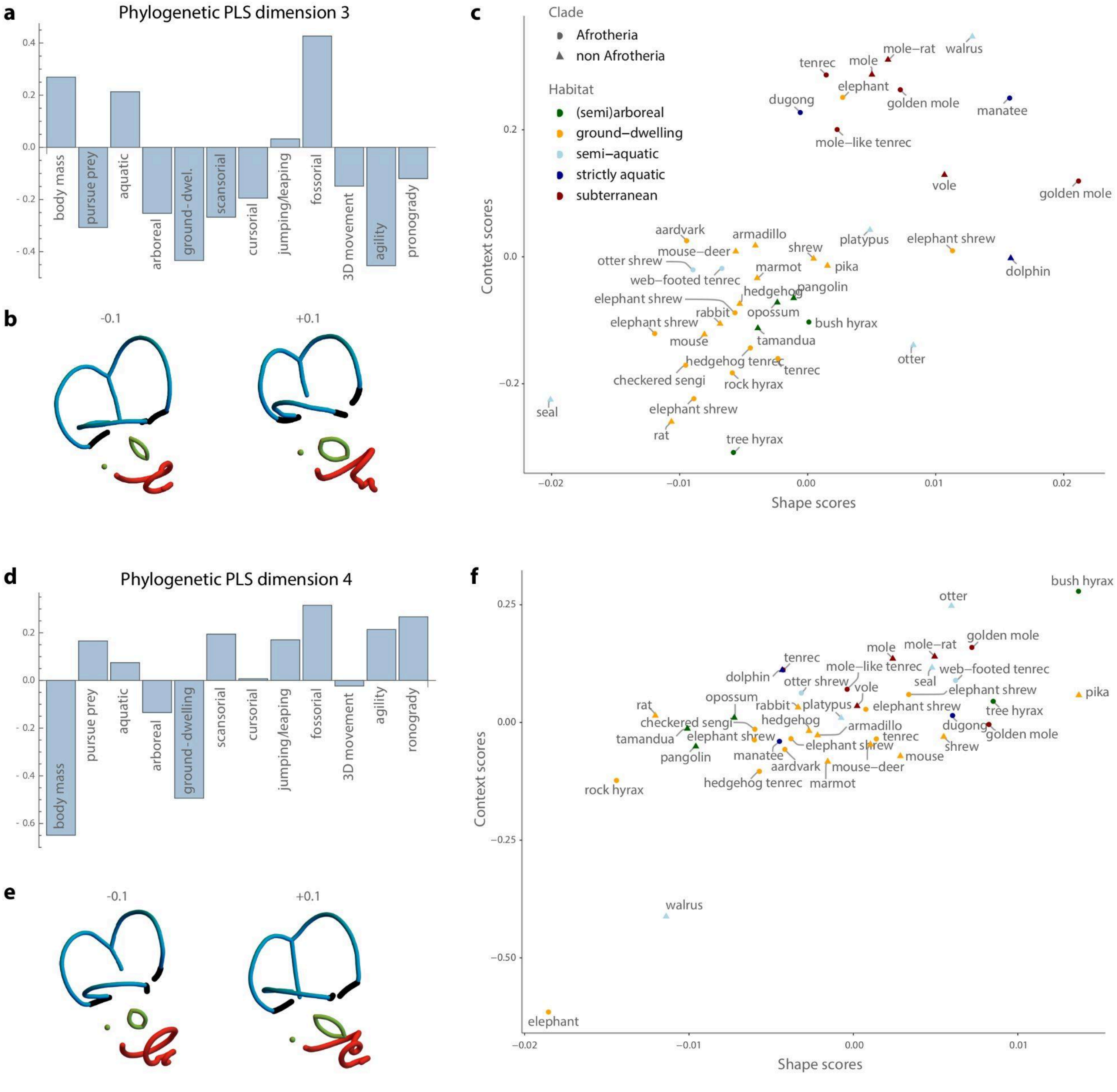

Supplementary Figure 4: Results for the phylogenetic partial least squares analysis of bony labyrinth shape and the contextual variables (PLS 3 and 4). Third and fourth dimensions of the phylogenetic two-block partial least squares (2B-PLS) analysis between the Procrustes shape coordinates of the bony labyrinth and the 12 contextual variables. **a** and **d** Loadings of the contextual variables for the third and fourth PLS dimensions, respectively. **b** and **e** Shape patterns corresponding to the loadings of the third and fourth PLS dimensions. The semicircular canals are in blue, the cochlea is in red, and the vestibular (oval) and cochlear (point) windows are in green. The orientation of the bony labyrinths are the same as in Fig. 2b. **c** and **f** Scatterplots of the corresponding contextual and shape scores for PLS 3 and PLS 4. The colour code corresponds to five habitat types which were

assigned to species only as a visual aid; this grouping was not used for computing the PLS. Note that when a species' common name appears several times, it corresponds to different species. Source data is provided in the Source Data file.

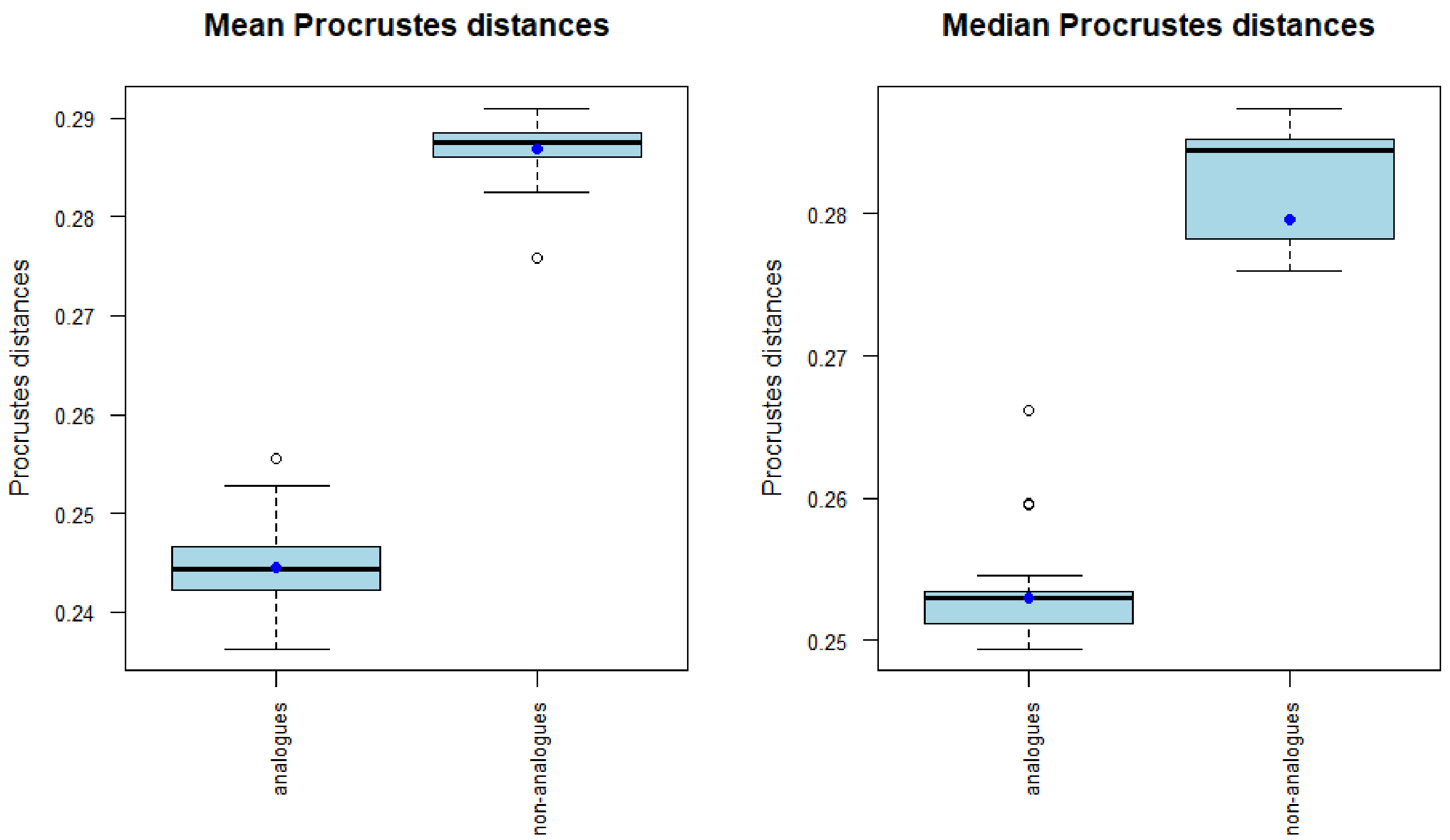

Supplementary Figure 5: Box plot showing the mean (left) and median (right) pairwise Procrustes distance between Afrotheria and non-afrotherian species for the full sample and the 39 leave-one-out samples. The box represents the interquartile range (IQR), i.e. the difference between the first quartile (Q1) and the third quartile (Q3). The bold line that separates the box into two parts is the median. The whiskers extend to the most extreme data point (excluding outliers), which is no more than the interquartile range times 1.5. Circles represent outliers, i.e. values beyond the limits of the whiskers. The blue dot corresponds to the average distance computed based on the full sample of 40 species. Source data is provided in the Source Data file.

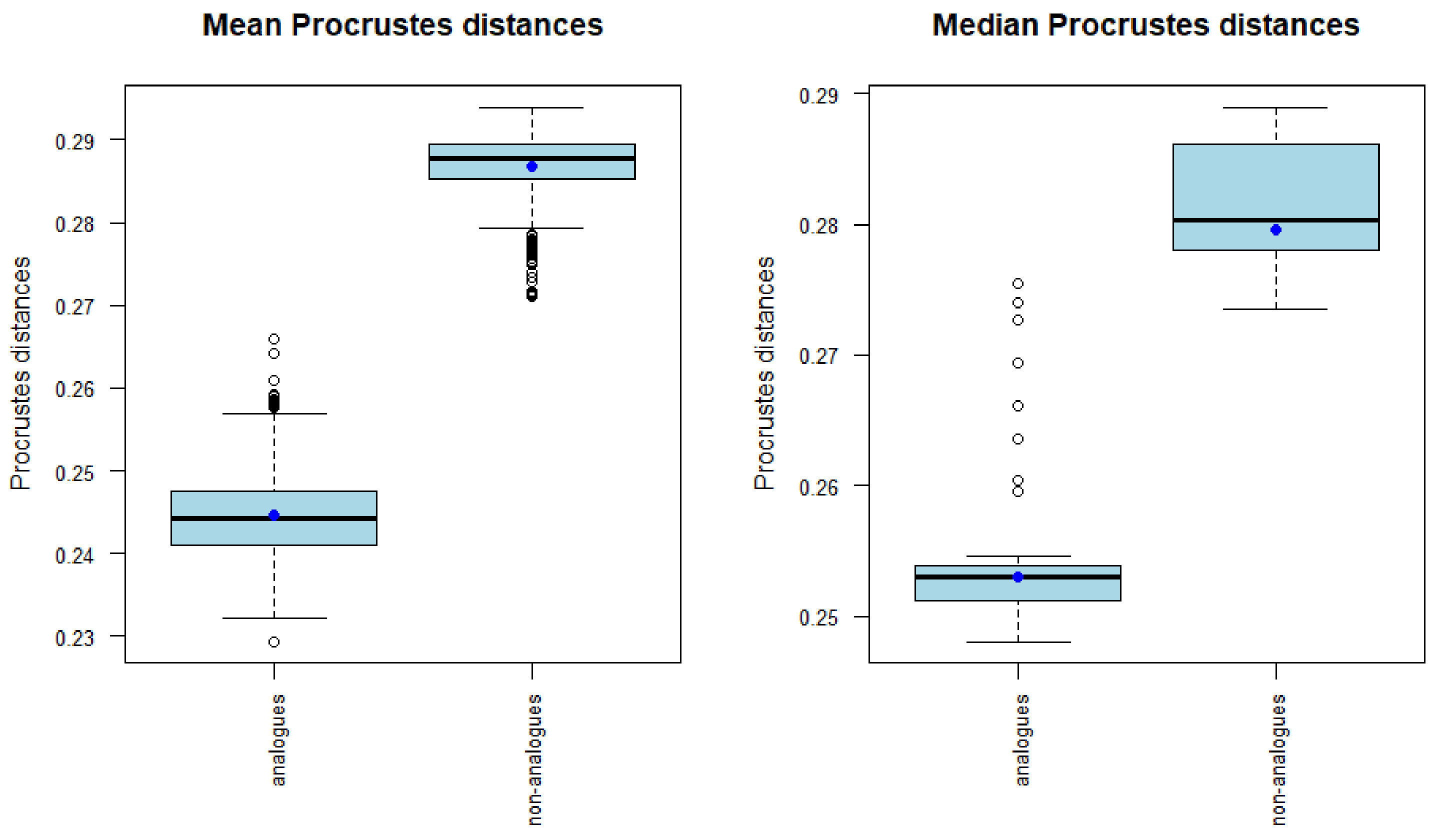

Supplementary Figure 6: Box plot showing the mean (left) and median (right) pairwise Procrustes distance between Afrotheria and non-afrotherian species for the full sample and the 780 leave-two-out samples. The box represents the interquartile range (IQR), i.e. the difference between the first quartile (Q1) and the third quartile (Q3. The bold line separating the box into two parts is the median. The whiskers extend to the most extreme data point (excluding outliers), which is no more than the interquartile range times 1.5. Circles represent outliers, i.e. values beyond the limits of the whiskers. The blue dot corresponds to the average distance computed for the whole sample of 40 species. Despite some overlap of the distributions of median Procrutes distances, within each replicate the median Procrustes distance between non-analogues always exceeded that between analogues. Source data is provided in the Source Data file.

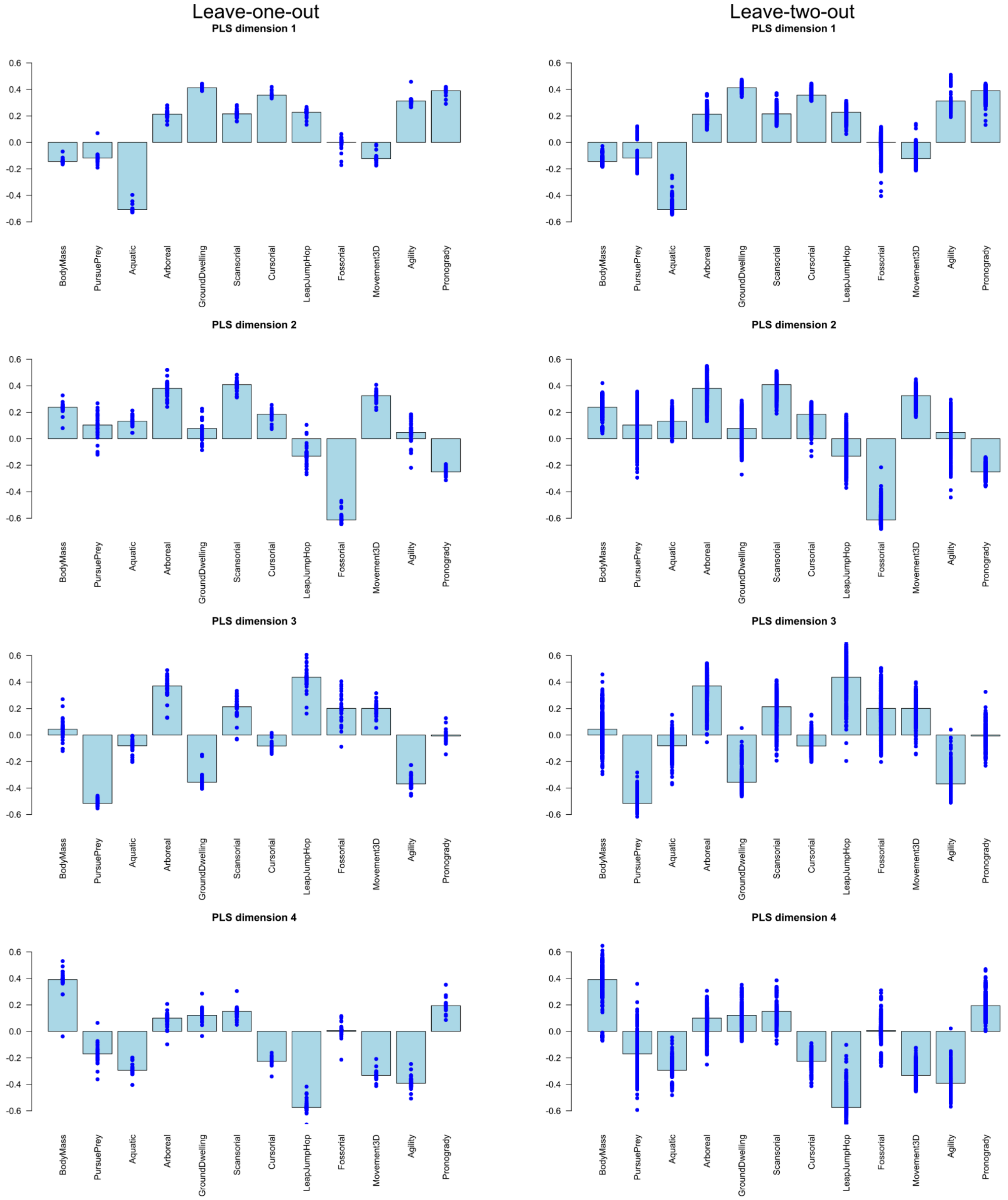

Supplementary Figure 7: Loadings of the contextual variables for the first four dimensions of the cross-validated two-block partial least squares (2B-PLS) analysis. Shown are the loadings of the contextual variables for the first four dimensions of the PLS analysis between the Procrustes shape coordinates of the bony labyrinth and the 12 contextual variables from our cross-validations. The bars show the loadings for the whole sample (*N*=40), while the points in the left panel correspond to the 40 replicates with one species removed each, and the points in the right panel correspond to the 780 replicates with two species removed. Source data is provided in the Source Data file.

Supplementary Tables

Supplementary Table 1. Explanation of the different pairwise combinations for which Procrustes distances in bony labyrinth shape are computed, using a few example afrotherians (see also Table 1 and Fig. 1 in the main text).

**Afrotherian**	**Non-afrotherian analogues**	**Non-afrotherian non-analogues**	**Other Afrotheria**
Potamogale velox	*Lutra lutra*	*Cryptomys hottentotus*	*Amblysomus hottentotus*
	Ornithorhynchus anatinus	*Talpa europaea*	*Chrysochloris asiatica*
		… (16 other non-afrotherians)	*Orizoryctes tetradactylus*
			… (16 remaining afrotherians)
Amblysomus hottentotus	*Cryptomys hottentotus*	*Lutra lutra*	*C. asiatica*
	Talpa europaea	*Ornithorhynchus anatinus*	*P. velox*
		… (16 other non-afrotherians)	*O. tetradactylus*
			… (16 remaining afrotherians)
Chrysochloris asiatica	As for *A. hottentotus* (see Table 1)	As for *A. hottentotus* (see Table 1)	*A. hottentotus*
			P. velox
			O. tetradactylus
			… (16 remaining afrotherians)
Orizoryctes tetradactylus	*T. europaea*	*L. lutra*	*P. velox*
		O. anatinus	*A. hottentotus*
		C. hottentotus	*C. asiatica*
		… (16 other non-afrotherians)	… (16 remaining afrotherians)
… (14 remaining afrotherians)	…	…	…

Supplementary Notes

Supplementary Note 1: Additional details on the computation of pairwise Procrustes distances

Supplementary Table 1 shows that *Potamogale velox* yields a total of two pairs with its analogues, a further 18 pairs with non-afrotherian non-analogues and, finally, 19 pairs with other afrotherians. *Amblysomus hottentotus* and *Chrysochloris asiatica* each also yield two analogue pairs, 18 non-analogue pairs, and 19 Afrotheria pairs. The same applies to *Orizoryctes tetradactylus.* The same procedure also applies to the 15 remaining afrotherians not included in Supplementary Table 1 (see **Table 1** in the main text for a full list of afrotherians and their analogues). Each non-afrotherian species (e.g. *Cryptomus hottentotus* or *Talpa europaea*) yielded 19 pairs with other non-afrotherians. Only unique pairwise distances were used in the analysis, i.e., *P. velox - A. hottentotus* is the same as *A. hottentotus - P. velox*.

Supplementary Note 2: Principal Component Analysis (PCA)

The first four principal components (PCs) accounted for 77.3% of total shape variance. Variation along PC 1 (26.9% of variance) corresponded to the size of the semicircular canals (SCC) relative to the cochlea (**Supplementary Fig. 1**). PC 1 and 2 combined mainly distinguish between the strictly aquatic species and the rest, showing convergence between the dolphin and the sea cows (manatee and dugong). Bony labyrinth shape features corresponding to an aquatic lifestyle include relatively small semicircular canals, a broader cochlea with a reduced number of turns, as well as a larger and rather round vestibular window. The highly specialist subterranean taxa, such as true moles, golden moles and certain tenrecs, cluster together on PC 3 and 4, another example of convergent evolution (**Supplementary Fig. 2**). Shape features of the bony labyrinth associated with a predominantly subterranean lifestyle are a relatively longer common crus, larger vertical semicircular canals (VSC) combined with a smaller lateral semicircular canal (LSC), and a cochlea with more turns than average. Arboreal taxa also tend to converge in labyrinth shape despite being phylogenetically distantly related (with the exception of the tree hyrax and bush hyrax), as evinced by their similarity in PC scores along all of the first four PCs. Bony labyrinth shape in tree-dwelling taxa includes a relatively shorter common crus, smaller and horizontally elongated VSC, a rounder LSC, and a cochlea with a broad base and fewer turns. On the other hand, ground-dwelling taxa (i.e., terrestrial species that are mostly on the ground) show a large range of ear shape variation, which is congruent with the diversity in ground-dwelling behaviours and lifestyles.

Overall, there are no obvious distinctions between afrotherians versus the rest, nor between the platypus (monotreme), opossum (marsupial), and placentals. Phylogenetic signal therefore does not appear to be strong (relative to the ecomorphological/functional signal) in labyrinth shape at the level of higher-order taxa.

Supplementary Note 3: Phylogenetic partial least squares

In addition to the 2B-PLS analysis in the main text, we conducted a phylogenetic 2B-PLS analysis to assess whether the associations between bony labyrinth shape and the contextual variables were mediated through the species' phylogenetic relationships. We found that similar associations remain after accounting for phylogeny, though the ecomorphological associations are captured by the four PLS dimensions in slightly different ways.

The first four dimensions together accounted for 90.0% of the summed squared covariances between the two blocks of variables (hierarchical permutation tests against a null hypothesis of no association yielded *p*-values of 0.02, 0.009, 0.054, and 0.088, respectively, for the four dimensions). Further dimensions accounted for only 2% or less. "Phylogenetic" PLS 1 (36.9% of the summed squared covariances) contrasted aquatic species from terrestrial species (**Supplementary Fig. 3**). Aquatic and semi-aquatic species had smaller and rounder semicircular canals associated with a larger, rounder vestibular window, and a broad and flat cochlea with fewer turns. Phylogenetic PLS 2 (27.2% of the summed squared covariances) distinguished arboreal and scansorial taxa from species that were more likely to pursue moving prey, be it in water or on the ground (**Supplementary Fig. 3**). The latter had a longer common crus, nearly as high as the vertical (anterior and posterior) semicircular canals, as well as a broader cochlea compared to arboreal and scansorial species.

Phylogenetic PLS 3 (16.6% of the summed squared covariances) contrasted agile species that pursue prey on the ground - and to some extent also agile aquatic species (especially the seal) - with species scoring low on agility (speed and manoeuvrability), i.e. subterraneous, fossorial as well as large-bodied taxa (**Supplementary Fig. 4**). Along this dimension, species' bony labyrinth shape mainly varied in the relative expansion of the semicircular canals and in the shape of the cochlear canal. Phylogenetic PLS 4 (9.3% of the summed squared covariances) was a contrast between slow and to some extent large-bodied species (driven by the elephant and the walrus) on the one hand and agile or fossorial species on the other (**Supplementary Fig. 4**). Along this dimension, species varied in the relative length of the common crus, the relative size and shape of the vestibular window, and in cochlear canal shape.

In comparison to the original PLS (see main text), then, the association between labyrinth shape and the eco-locomotor variables along PLS 1 stays roughly the same, i.e. a contrast between aquatic vs. more ground-dwelling, pronograde species, incl. a similar labyrinth shape pattern. Consecutive PLS dimensions also remain similar, though the association with particular ecologies (e.g. fossoriality) are sometimes differently distributed across the PLS dimensions. For instance, in the phylogenetic PLS fossoriality is associated with a similar labyrinth shape as before, but along PLS 3 instead of PLS 2.

Supplementary Note 4: Jackknife of the Procrustes distances

We computed all the pairwise Procrustes distances between Afrotheria and non-afrotherian species, and compared the average distance (expressed as the arithmetic mean or as the median) between the

pairs of analogues (i.e., between each afrotherian species and its non-afrotherian analogue) and the pairs of non-analogues (i.e., between each afrotherian species and its non-afrotherian non-analogue). Because the elephant (*Elephas maximus*) does not have an analogue in our study, we excluded it from these computations. For the full sample of 40 species, the mean Procrustes distance between afrotherians and their analogues was significantly smaller than the average Procrustes distance between afrotherians and non-analogues, supporting our hypothesis of convergent evolution (see main text). Here we show that both for the mean Procrustes distance and for the median Procrustes distance, this result was replicated for all 39 leave-one-out (jackknife) samples (**Supplementary Fig. 5**) and for the 780 leave-two-out samples (**Supplementary Fig. 6**).

Also, when leaving out complete analogue pairs instead of single species, the average Procrustes distance between analogues was always smaller than the average Procrustes distance between non-analogues (the ratio of average distances ranged from 1.14 to 1.20 when leaving out one and two analogue pairs, respectively).

Supplementary Note 5: *K*-fold cross-validation of the PLS analyses

We performed a two-block partial least squares (2B-PLS) analysis between the Procrustes-aligned coordinates and the 12 standardised contextual variables for the entire sample of 20 afrotherian species and 20 non-afrotherian species (see main text). We tested if our results were stable despite the limited sample size and the high morphological and ecological disparity. To this end, we performed a leave-one-out cross-validation, also known as jackknife resampling (i.e., the PLS analysis is repeated 40 times with one species left out each time) and a leave-two-out cross-validation (40x39/2=780 PLS analyses with two species left out each time). Because of the specific, non-random sample composition and the relatively small sample size, we prefer this approach over bootstrap resampling. Note that for each iteration we removed the *K* (1 or 2) species only at the 2B-PLS step (i.e., we used the Procrustes coordinates and contextual variables that were aligned or standardised using the whole sample of all 40 species).

The cross-validations showed that results of the 2B-PLS analyses are quite stable when one and even when two species are removed (**Supplementary Fig. 7**). The same PLS patterns are recovered as when using the total sample (see main text):

- PLS 1: aquatic *vs.* ground-dwelling, cursorial, pronograde or agile species
- PLS 2: fossorial and pronograde vs. arboreal, scansorial and species moving in 3D
- PLS 3: agile species pursuing prey on the ground *vs.* arboreal species that leap or jump
- PLS4: the signal is less stable but the results are still consistent for leave-one-out cross-validation: heavy species *vs.* agile species moving in a three-dimensional space – arboreal or aquatic.
