# Supplementary material for: Convergent evolution in Afrotheria and non-afrotherians demonstrates high evolvability of the mammalian inner ear
Source: Nat Commun. 2024 Sep 16;15:7869. doi: 10.1038/s41467-024-52180-1 (PMC11405882; doi:10.1038/s41467-024-52180-1)
Supplement: Supplementary file 3 — Reporting Summary [file 41467_2024_52180_MOESM3_ESM.pdf]

nature portfolio

Corresponding author(s): Nicole D.S. Grunstra, Anne Le Maître

Last updated by author(s): Jul 25, 2024

Reporting Summary

Nature Portfolio wishes to improve the reproducibility of the work that we publish. This form provides structure for consistency and transparency in reporting. For further information on Nature Portfolio policies, see our Editorial Policies and the Editorial Policy Checklist.

Statistics

For all statistical analyses, confirm that the following items are present in the figure legend, table legend, main text, or Methods section.

n/a	Confirmed	
☐	☒	The exact sample size (*n*) for each experimental group/condition, given as a discrete number and unit of measurement
☐	☒	A statement on whether measurements were taken from distinct samples or whether the same sample was measured repeatedly
☐	☒	The statistical test(s) used AND whether they are one- or two-sided<br>*Only common tests should be described solely by name; describe more complex techniques in the Methods section.*
☐	☒	A description of all covariates tested
☐	☒	A description of any assumptions or corrections, such as tests of normality and adjustment for multiple comparisons
☐	☒	A full description of the statistical parameters including central tendency (e.g. means) or other basic estimates (e.g. regression coefficient) AND variation (e.g. standard deviation) or associated estimates of uncertainty (e.g. confidence intervals)
☐	☒	For null hypothesis testing, the test statistic (e.g. *F*, *t*, *r*) with confidence intervals, effect sizes, degrees of freedom and *P* value noted<br>*Give P values as exact values whenever suitable.*
☒	☐	For Bayesian analysis, information on the choice of priors and Markov chain Monte Carlo settings
☒	☐	For hierarchical and complex designs, identification of the appropriate level for tests and full reporting of outcomes
☒	☐	Estimates of effect sizes (e.g. Cohen's *d*, Pearson's *r*), indicating how they were calculated

Our web collection on statistics for biologists contains articles on many of the points above.

Software and code

Policy information about availability of computer code

Data collection	We virtually segmented the bony labyrinth from CT-scans and placed the landmarks using the software Amira (Thermo Fisher Scientific) version 2020.2
Data analysis	All analyses were performed in R (R Development Core Team, 2023, version 4.3.1) and in Wolfram Mathematica 12, with nearly identical results. Results presented in the text were obtained in R. R scripts are provided in GitHub (https://github.com/dalemaitre/Afrotheria) and in Zenodo (https://zenodo.org/records/12749955) and linked to the OSF online repository (https://osf.io/9mtwh/). We used the R packages ape 5.7-1 (Paradis & Schliep, 2019), geomorph 4.0.6 (Baken et al., 2021, Adams et al., 2023), Morpho 2.11 (Schlager, 2017) and phytools 2.0-3 (Revell, 2012). We generated scatter plots using the packages ggplot2 3.4.4 (Wickham, 2016) and ggrepel 0.9.4 (Slowikowski, 2023). Visualisations of the shape changes and contextual loadings were generated in Mathematica.

For manuscripts utilizing custom algorithms or software that are central to the research but not yet described in published literature, software must be made available to editors and reviewers. We strongly encourage code deposition in a community repository (e.g. GitHub). See the Nature Portfolio guidelines for submitting code & software for further information.

Data

Policy information about availability of data

All manuscripts must include a data availability statement. This statement should provide the following information, where applicable:

- Accession codes, unique identifiers, or web links for publicly available datasets
- A description of any restrictions on data availability
- For clinical datasets or third party data, please ensure that the statement adheres to our policy

All 3D surface models, the raw and slid landmark coordinates (not Procrustes-aligned), as well as the detailed sample composition, the table of contextual variables and the ultrametric phylogenetic tree are available and freely accessible in the OSF repository [https://osf.io/9mtwh/]. Source data for Figures 3, 4, and for all Supplementary Figures are provided as a single Source Data file.
Supplementary Text, including supplementary Tables and Figures, was uploaded directly as a single document associated with the main text to the journal website.
All R code and raw data files required to reproduce the analyses are available as supplementary data files in a GitHub repository [https://github.com/dalemaitre/Afrotheria], also available on Zenodo [https://zenodo.org/records/12749955] and in the paper's OSF repository [https://osf.io/9mtwh/].

Research involving human participants, their data, or biological material

Policy information about studies with human participants or human data. See also policy information about sex, gender (identity/presentation), and sexual orientation and race, ethnicity and racism.

Reporting on sex and gender	n/a
Reporting on race, ethnicity, or other socially relevant groupings	n/a
Population characteristics	n/a
Recruitment	n/a
Ethics oversight	n/a

Note that full information on the approval of the study protocol must also be provided in the manuscript.

Field-specific reporting

Please select the one below that is the best fit for your research. If you are not sure, read the appropriate sections before making your selection.

☐ Life sciences ☐ Behavioural & social sciences ☒ Ecological, evolutionary & environmental sciences

For a reference copy of the document with all sections, see nature.com/documents/nr-reporting-summary-flat.pdf

Ecological, evolutionary & environmental sciences study design

All studies must disclose on these points even when the disclosure is negative.

Study description

We study convergent evolution in the bony labyrinth (osseous surroundings of the inner ear) between afrotherian and non-afrotherian mammals which are their morphological, functional or ecological analogues. For this, we describe the morphology of the bony labyrinth by a comprehensive set of 13 landmarks and 111 semilandmarks placed on the midline and the windows of the labyrinth. We evaluate the association between labyrinth shape and 12 contextual variables of body mass, ecology and positional behaviour, using two-block partial least square analyses (2B-PLS).
To further undersand the results, we performed other analyses:
- a test of the phylogenetic signal in labyrinth shape (to test for phylogenetic convergence or divergence),
- a phylogenetic 2B-PLS between labyrinth shape and contextual variables (to correct for the phylogeny),
- a principle component analysis of labyrinth shape (to explore shape variation across all taxa),
- a comparison of pairwise Procrustes distances among analogues and among non-analogues (to see if analogues are more similar), as well as among Afrotheria and among non-afrotherian mammals
- a computation of the "Wheatsheaf index", which is the ratio of the average Procrustes distance between pairs of analogues to the average Procrustes distance between all species while statistically correcting for phylogenetic relatedness

Research sample

The sample corresponds to 20 Afrotheria and 20 non-afrotherian mammals. We selected Afrotheria for our study of convergent evolution in the bony labyrinth, beecause of their huge phenetic, ecological, behavioural and morphological disparity, and the fact they are known for convergent evolution in many anatomical features.
The 20 non-afrotherians comprise: 1 Monotremata, 1 Marsupialia, 2 Xenarthra, 7 Euarchontoglires, 9 Laurasiatheria, all of which are analogues to at least one afrotherian taxa.
There are 9 females, 13 males, and 18 specimens of unknown sex, but this has no impact in our study because sexual dimorphism is very low for the bony labyrinth. Some specimens are not adults (at least 4), which is not a problem because the bony labyrinth reaches its adult size and shape before birth.

Sampling strategy	We first selected 20 Afrotheria taxa to have a sufficient diversity within this group. We tried to choose Afrotheria representing the widest possible taxonomic phenetic, ecological, behavioural and morphological disparity. Then, we selected the same number of analogues species within non-afrotherian mammals, mostly placental mammals, and a marsupial and a monotreme as phylogenetic outgroups.<br>To assess the robustness of the results to changes in sample composition, we performed leave-one-out and leave-two-out cross-validations of the 2B-PLS analyses and the Procrustes distances. All the replicates led to results very similar to those obtained with the total sampel and support the same interpretation of the data.
Data collection	The CT-scans were downloaded (n = 13) from the online repository MorphoSource (www.MorphoSource.org, Duke University) or performed by Cathrin Pfaff (n = 17) or Viola Winkler (n = 10).<br>The segmentation of the bony labyrinth was done by three of us (Cathrin Pfaff, Guillermo Bravo and Anne Le Maître), using the segmentation tools in the software Amira (brush, wagic wand, selection by threshold).<br>The landmark data was collected by Fabian Hollinetz using Amira, based on the reconstruction of the centerlines using the AutoSkeleton module, and Anne Le Maître had a final look to check the landmark placement.<br>Any inter-individual differences in segmentation are likely to be very minor and have no effect on the final data, because landmarks were not placed in anatomical parts sensitive to these differences.<br>Contextual data were collected by Fabian Hollinetz and Nicole Grunstra in an Excel sheet.<br>Nicole Grunstra also collected the phylogenetic tree from the website www.VertLife.org.
Timing and spatial scale	Landmark data collection: form 2021-11-23 to 2022-10-17, most of it collected from 2022-04-20 to 2022-05-04.<br>Final check of landmark data for all specimens and correction on 2023-02-23 and 2023-02-24.
Data exclusions	No data were excluded from the analyses.
Reproducibility	The analyses presented in the manuscript are performed in Mathematika using a custom script. Independently (a different person realized the analyses), we reproduced the analyses in R using the build-in functions for geometric morphometrics (hence a different script doing the same steps), and we found exactly the same results. We provide all R scripts.
Randomization	We did not randomize our sample. On the contrary, we choos the non-afrotherian sample so that each taxa is analogue to at least on Afrotheria taxa.
Blinding	Blinding was not relevant to our study, because the way we test for convergent evolution requires to know each taxa and its clade.

Did the study involve field work? ☐ Yes ☒ No

Reporting for specific materials, systems and methods

We require information from authors about some types of materials, experimental systems and methods used in many studies. Here, indicate whether each material, system or method listed is relevant to your study. If you are not sure if a list item applies to your research, read the appropriate section before selecting a response.

Materials & experimental systems

n/a	Involved in the study
☒	☐ Antibodies
☒	☐ Eukaryotic cell lines
☒	☐ Palaeontology and archaeology
☐	☒ Animals and other organisms
☒	☐ Clinical data
☒	☐ Dual use research of concern
☒	☐ Plants

Methods

n/a	Involved in the study
☒	☐ ChIP-seq
☒	☐ Flow cytometry
☒	☐ MRI-based neuroimaging

Animals and other research organisms

Policy information about studies involving animals; ARRIVE guidelines recommended for reporting animal research, and Sex and Gender in Research

Laboratory animals	The study did not involve laboratory animals
Wild animals	The study did not involve wild animals
Reporting on sex	The sample comprises 9 females (5 Afrotheria vs. 4 non-afrotherian), 13 males (5 vs. 8), and 18 (10 vs. 8) specimens of unknown sex. Because the bony labyrinth is only slightly sexually dimorphic, and the focus of our study was interspecific differences, which are likely to be much higher than within-species differences, we did not consider sexual dimorphism.
Field-collected samples	The sample is composed of museum specimens from historical collections, hence did not involve sample collected from the field by us. The detailed provenance (museum, country of origin when available) is provided as supplementary data in an online repository

(see above).

Ethics oversight	No ethical approval was required, because we used specimens from historical collections in museums.

Note that full information on the approval of the study protocol must also be provided in the manuscript.
